# Association between postoperative changes in the gut microbiota and pseudopsia after cardiac surgery: prospective observational study

**DOI:** 10.1186/s12893-020-00907-4

**Published:** 2020-10-20

**Authors:** Masaki Maekawa, Kenji Yoshitani, Musashi Yahagi, Takashi Asahara, Yoshiyuki Shishido, Satsuki Fukushima, Naoki Tadokoro, Tomoyuki Fujita, Yoshihiko Ohnishi

**Affiliations:** 1 Department of Anesthesiology, National Cerebral and Cardiovascular Center, Suita, Japan; 2 Yakult Central Institute, Kunitachi, Japan; 3 Department of Cardiovascular Surgery, National Cerebral and Cardiovascular Center, Suita, Japan

**Keywords:** Delirium, Microbiota, Gut–brain axis, Cardiac surgery

## Abstract

**Background:**

Delirium after cardiac surgery affects mortality, but the mechanism remains unclear. Previous studies have reported gut microbiota are associated with brain activity. Systemic inflammation and antibiotics can damage the gut microbiota after cardiac surgery. We aimed to investigate changes in the gut microbiota and the association between the gut microbiota and delirium after cardiac surgery.

**Methods:**

Twenty-one patients who underwent cardiac surgery were enrolled. Microbiota counts and fecal organic acid concentrations were measured in fecal samples harvested before surgery, just after surgery, and before discharge. To quantify the microbiota, we extracted total RNA fractions and examined gut microbiota composition using 16S and 23S rRNA-targeted quantitative-reverse Transcription-PCR. Postoperative delirium, insomnia, and pseudopsia were assessed for 1 week. Postoperative total bacterial counts changed significantly from 10.2 ± 0.2 log_10_ cells/g of feces to 9.8 ± 0.5 in the first postoperative samples (*p* = 0.003) and 10.0 ± 0.4 in the samples before discharge (*p* = 0.039). Fecal pH was 6.9 ± 0.6 before surgery and 7.4 ± 0.7 in the first postoperative samples (*p* = 0.001). Postoperative *Staphylococcus* and *Pseudomonas* counts were significantly higher in patients with postoperative pseudopsia than in patients without pseudopsia (3.2 ± 1.3 vs. 5.4 ± 0.9; *p* = 0.012 and 1.7 ± 0.8 vs. 4.6 ± 2.7; *p* = 0.001).

**Conclusions:**

Total bacterial counts were significantly lower after surgery and until discharge. Fecal pH was significantly higher than preoperative levels. *Staphylococcus* and *Pseudomonas* counts were significantly higher in patients with postoperative pseudopsia.

## Background

Postoperative delirium after cardiac surgery is a serious complication and an independent predictor of worse prognosis [1]. Some studies have reported risk factors for delirium, such as age and inflammation [2, 3]. However, details about postoperative delirium remain to be clarified.

Previous studies have demonstrated that individual differences in the gut microbiota influence health status [4, 5] as well as brain activity, which is referred to as microbiota–gut–brain communication [6, 7]. Patients who undergo cardiac surgery with cardiopulmonary bypass who develop systemic inflammation [8] and receive multiple drugs experience damage to their gut flora [9, 10]. Some studies claim that damaged gut flora may lead to perioperative complications [11]. We hypothesize that postoperative delirium can be associated with damage to gut flora.

This single-center prospective observational study aimed to identify changes in gut flora after cardiopulmonary bypass and differences in gut flora between patients with and without postoperative pseudopsia or insomnia, which are symptoms of postoperative delirium.

## Methods

### Study design

This study was a single-center prospective observational study to investigate perioperative changes in the gut microbiota associated with cardiac surgery and the association between gut flora and postoperative pseudopsia or insomnia. The study was approved by the institutional ethics committee and conforms to the ethical norms and standards in the Declaration of Helsinki.

### Patients

In this study, 21 adult patients who underwent elective cardiac surgery with cardiopulmonary bypass were enrolled. Participants provided written informed consent. Exclusion criteria included another major illness except for preoperative hypertension, diabetes mellitus, or dyslipidemia; selective cerebral perfusion; deep hypothermic circulatory arrest; aortic surgery; and preoperative administration of antibiotics. Preoperative and perioperative patient characteristics were collected from medical records.

### Delirium assessment

Postoperative delirium was assessed using the CAM-ICU scale (https://www.icudelirium.org/medical-professionals/delirium/monitoring-delirium-in-the-icu) during the week after extubation, which has been used most frequently [12, 13]. Furthermore, postoperative pseudopsia and insomnia were investigated simultaneously. They are not included in the CAM-ICU, but are included in the Diagnostic and Statistical Manual of Mental Disorders, 5th edition. Symptoms of delirium were also included. Pseudopsia and insomnia were assessed by Intensive Care Unit (ICU) physicians. The insomnia group included patients who had insomnia for more than 2 days after surgery to exclude the effects of general anesthesia. The pseudopsia group contained patients who had pseudopsia on more than one occasion.

### Anesthetic management

All patients underwent induction of general anesthesia with 0.5–1 mg/kg of midazolam, 2–10 μg/kg of fentanyl, and 0.6–1 mg/kg of rocuronium. Anesthesia was maintained with 4–6 mg/kg/h of propofol, 30 μg/kg/h of remifentanil, and 0.4–0.5 mg/kg/h of rocuronium. Sevoflurane was sometimes added in order to control blood pressure for short periods of time. Local anesthesia was not always used. All patients received 3 g/day of cefazolin on postoperative day (POD) 0 and 1.

### Fecal sample collection

Patients collected their own fecal samples at three time points. The first sample, which served as the control, was harvested a few days before surgery. The second sample was from the first or second bowel movement after surgery. In general, the first postoperative sample was harvested but some patients were critically ill and the second sample was harvested. The last sample was harvested sometime between (POD) 6 and 8. Patients placed the fecal samples directly into two tubes (approximately 1.0 g/tube). One tube contained 2 mL RNAlater (an RNA stabilization solution; Ambion, Austin, TX, USA), and the other was empty. The samples with RNAlater were placed in a refrigerator at 4 °C for analysis of the fecal microbiota. The other samples were placed in a freezer at − 80 °C within 30 min of excretion for analysis of fecal organic acid concentrations and pH. Samples were transported to the Yakult Central Institute at − 20 °C for analysis. The patient’s identity, clinical information, and study group were unknown to the technicians performing the analysis.

### Gut microbiota analysis

To quantify the bacteria present in the samples, we extracted total RNA fractions from the fecal samples using previously described methods [14–17]. We examined gut microbiota composition using 16S and 23S rRNA targeted quantitative Reverse Transcription PCR (qRT-PCR) using the Yakult Intestinal Flora-SCAN analysis system (YIF-SCAN^®^; Yakult Honsha, Tokyo, Japan) [16]. Three serial dilutions of each extracted RNA sample were used for rRNA-targeted qRT-PCR. Threshold cycle values in the linear range of the assay were applied to the standard curve to obtain the corresponding bacterial cell count for each fecal or blood sample. In this study, predominant anaerobes present in the human intestine (*Clostridium coccoides* group, *Clostridium leptum* subgroup, *Bacteroides fragilis* group, *Bifidobacterium*, *Atopobium* cluster, and *Prevotella*) and intestinal subdominant populations (*Clostridium difficile*, *Clostridium perfringens, Lactobacillus*, *Enterobacteriaceae*, *Enterococcus*, *Streptococcus, Staphylococcus,* and *Pseudomonas*) were examined. The specificity of the qRT-PCR assay using group-specific, genus-specific, and species-specific primers was determined as described previously [14, 15, 17–19].

### Short-chain fatty acids concentration (SCFAs) and pH measurement

Concentrations of fecal SCFAs were measured as described previously [20] with slight modifications. Briefly, the frozen samples were homogenized in four-fold volumes of 0.15 mol/l perchloric acid and allowed to stand at 4 °C for 12 h. The suspension was subjected to centrifugation at 20,400×*g* at 4 °C for 10 min. The resultant supernatant was passed through a filter with a pore size of 0.45 μm (Millipore Japan, Tokyo, Japan). The sample was analyzed for organic acids using a high-performance liquid chromatography system (Waters 432 Conductivity Detector, Waters, Milford, MA, USA). Organic acid concentrations were calculated with the use of external standards and expressed as µmol/g of wet feces. The lower limits for fecal organic acid concentrations using this procedure were 0.075 µmol/g for succinic acid, 0.2 µmol/g for lactic acid, 0.05 µmol/g for formic acid, 0.4 µmol/g for acetic acid, 0.5 µmol/g for propionic acid, 0.55 µmol/g for butyric acid, 0.8 µmol/g for isovaleric acid, and 0.65 µmol/g for valeric acid. Fecal pH was measured by directly inserting the glass electrode of a D-51 pH meter (Horiba Seisakusho, Tokyo, Japan) into a sample of homogenized feces.

### Statistical analysis

Postoperative microbiota counts and fecal organic acid concentrations were compared with preoperative control values using the paired t test. If microbiota counts were lower than the limit of detection, the count of the sample was treated as half of the limit of detection value. Moreover, microbiota counts and fecal organic acid concentrations were compared between patients with or without pseudopsia and insomnia using the unpaired t test. Analyses were performed using Stata/SE, version 16 (StataCorp, College Station, TX, USA).

## Results

The perioperative characteristics parameters of the 21 patients are shown in Table 1. There were no patients with delirium diagnosed based on the CAM-ICU. Three patients had pseudopsia and 11 patients had insomnia for more than 2 days. Consequently, we could not compare differences between patients with and without delirium diagnosed based on the CAM-ICU. No unexpected critical adverse events were observed perioperatively. All patients were extubated in the ICU; 20 of 21 patients were extubated on the day of surgery. Only one patient remained intubated until the next morning. All patients started oral intake and physical rehabilitation on POD 1. Cefazolin was used in 19 patients. Two patients received additional antibiotics because of suspected postoperative infection. In one patient, doripenem hydrate was added on POD 3. In another patient, vancomycin and meropenem hydrate were added on POD 6.

**Table 1:** Perioperative characteristics and parameters

	Number of patients (%) (n = 21)
Age (years), median (range)	62 (22–80)
Male sex, n (%)	16 (76.2)
Smoking status	
Never smoker	9 (42.9)
Previous smoker	10 (47.6)
Current smoker	2 (9.5)
Hypertension	11 (52.4)
Diabetes mellitus	3 (14.3)
Dyslipidemia	9 (42.9)
Operation, n (%)	
Valve surgery	17 (81.0)
Valve surgery + CABG	2 (9.5)
Tumor removal	1 (4.8)
Myectomy	1 (4.8)
Dairy-based ingestion of lactobacillus, n (%)	10 (47.6)
Postoperative delirium, n (%)	0 (0)
Postoperative pseudopsia, n (%)	3 (14.3)
Postoperative insomnia, n (%)	11 (52.4)

Valve surgery included aortic valve replacement (n = 9), mitral valve repair (n = 8), and mitral valve replacement (n = 2)

*CABG* coronary artery bypass grafting

On average, the first postoperative samples were harvested on POD 3.2. More specifically, samples were harvested on POD 2 in 3 patients, POD 3 in 12 patients, POD 4 in 4 patients, and POD 6 in 1 patient. One patient could not harvest the second sample (first postoperative sample). All analyses comparing preoperative versus first postoperative samples were based on the other 20 patients.

### Total microbiota counts and concentrations of SFCAs in feces

**Fig. 1:**
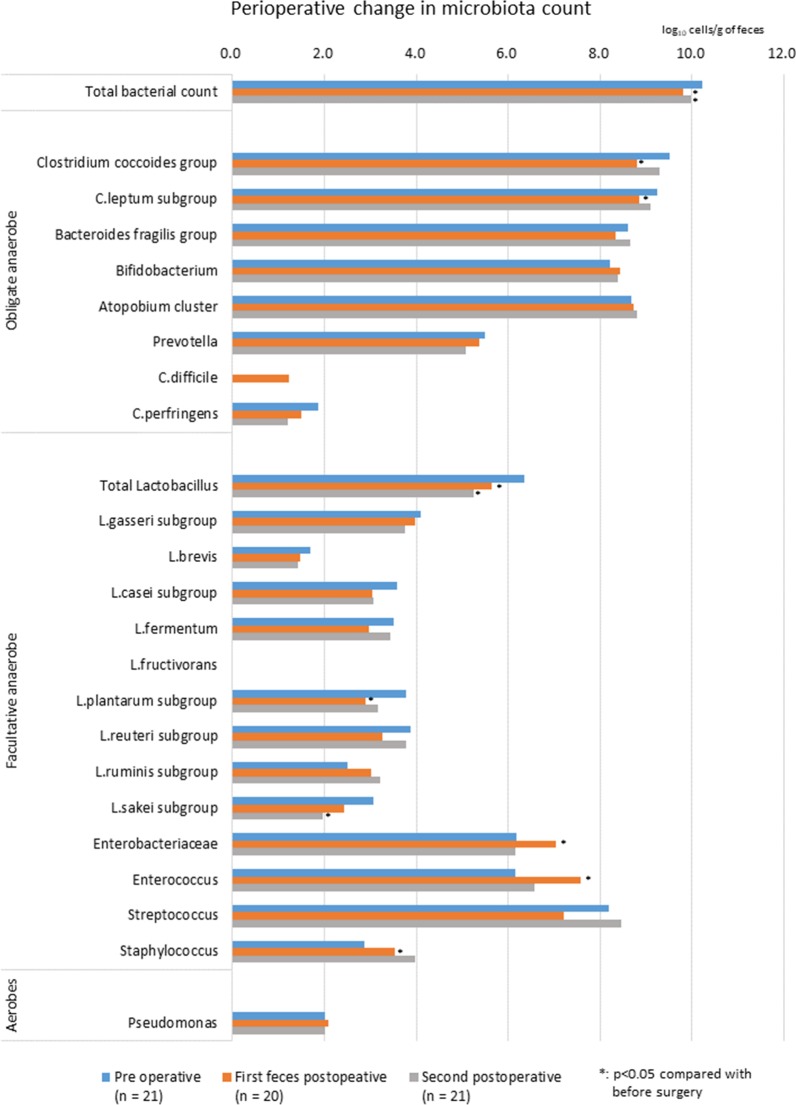
Perioperative changes in microbiota counts. Blue bars represent preoperative counts, orange bars represent first postoperative counts, and gray bars represent second postoperative counts (around POD 7). Each postoperative microbiota count was compared with the corresponding preoperative count with the paired t test. Asterisk shows significant differences. *POD* postoperative day

All values were compared with preoperative levels. Microbiota counts are presented as log_10_ cells/g of feces. Figure 1 shows the perioperative changes in microbiota count. Compared with preoperative total microbiota counts (10.2 ± 0.2), counts in the first and second postoperative fecal samples were significantly lower (9.8 ± 0.5; *p* = 0.003 and 10.0 ± 0.4; *p* = 0.039, respectively).

Among obligate anaerobes, *Clostridium coccoides* group counts were significantly lower in the first postoperative fecal samples than in preoperative samples (8.8 ± 0.7 vs. 9.5 ± 0.4; *p* = 0.001). *Clostridium leptum* subgroup counts were also lower in the first postoperative samples (8.9 ± 0.8 vs. 9.2 ± 0.8; *p* = 0.040).

Among facultative anaerobes, postoperative total *Lactobacillus* counts, both in the first postoperative and second postoperative samples (collected around POD 7), were significantly lower: preoperative; 6.4 ± 1.4; first postoperative, 5.6 ± 1.4 (*p* = 0.013); and second postoperative, 5.3 ± 2.4 (*p* = 0.025). Of note, *L. plantarum* subgroup counts were lower in the first postoperative fecal samples: preoperative, 3.8 ± 1.6 versus first postoperative, 2.9 ± 1.3 (*p* = 0.038). *L. sakei* subgroup counts were lower in the second postoperative samples collected around POD 7: preoperative, 3.1 ± 2.1 versus second postoperative, 2.0 ± 1.3 (*p* = 0.036).

By contrast, *Enterobacteriaceae* counts were significantly higher in the first postoperative fecal samples compared with preoperative samples (7.0 ± 0.7 vs. 6.2 ± 1.5, *p* = 0.031). *Enterococcus* counts were also significantly higher in the first postoperative fecal samples compared with preoperative samples (7.6 ± 2.0 vs. 6.2 ± 1.7; *p* = 0.009). *Staphylococcus* counts were significantly higher in the second postoperative fecal samples compared with preoperative samples (4.0 ± 1.2 vs. 2.9 ± 1.5; *p* = 0.002).

**Fig. 2:**
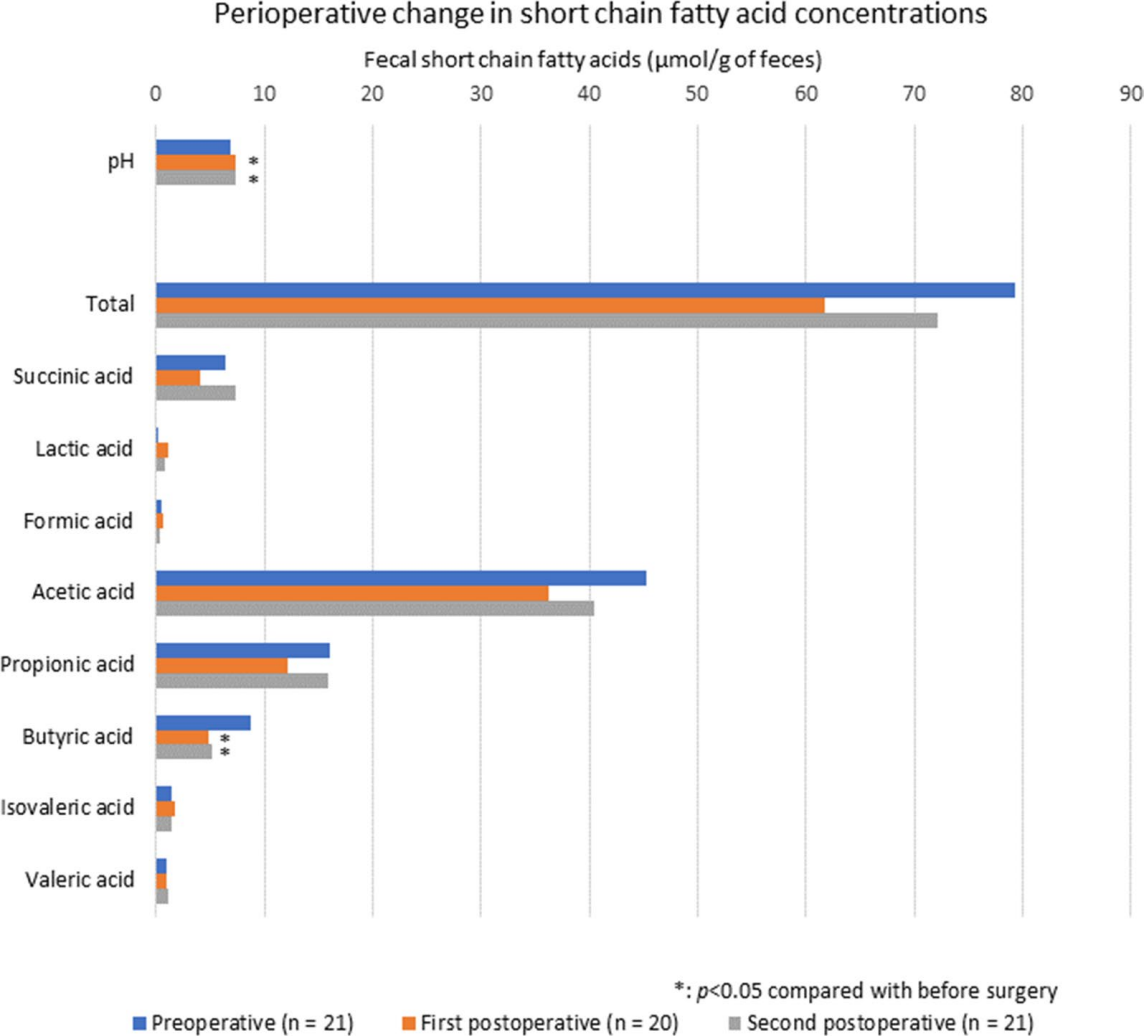
Perioperative changes in fecal pH and concentrations of short-chain fatty acids (SCFAs) in feces. Blue bars represent preoperative values, orange bars represent first postoperative values, and gray bars represent second postoperative values (around POD 7). Fecal pH and SCFAs concentrations in the first postoperative and second postoperative fecal examples were compared with preoperative values. Asterisk shows significant differences. *POD* postoperative day

Figure 2 shows perioperative changes in fecal SFCAs concentrations. The first postoperative and second postoperative fecal samples were significantly more alkalinized than preoperative samples: preoperative pH, 6.9 ± 0.6; first postoperative pH, 7.4 ± 0.7 (*p* = 0.011); and second postoperative pH, 7.4 ± 0.7 (*p* = 0.001). Postoperative butyric acid concentrations were lower after surgery: preoperative, 8.7 ± 5.9 μmol/g of feces; first postoperative, 4.9 ± 3.1 μmol/g (*p* = 0.026); second preoperative, 5.2 ± 5.5 μmol/g (*p* = 0.008).

### Microbiota counts and concentrations of fecal SFCAs in patients with and without pseudopsia

**Fig. 3:**
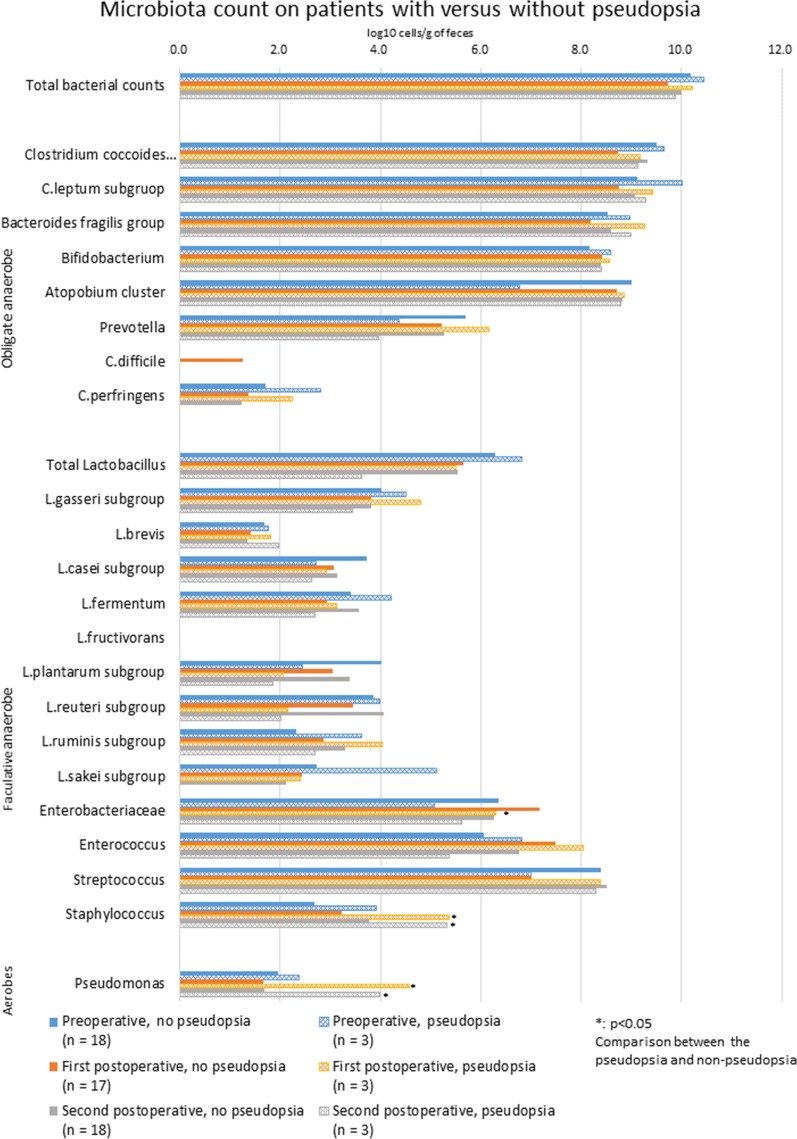
Perioperative changes in microbiota counts in patients with versus without pseudopsia. Blue bars represent preoperative counts, orange bars represent first postoperative counts, and gray bars represent second postoperative counts (around POD 7). The mesh pattern depicts the pseudopsia group. For each measurement timepoint, differences between patients with and without pseudopsia were compared using the t test. Asterisk shows significant differences. *POD* postoperative day

**Fig. 4:**
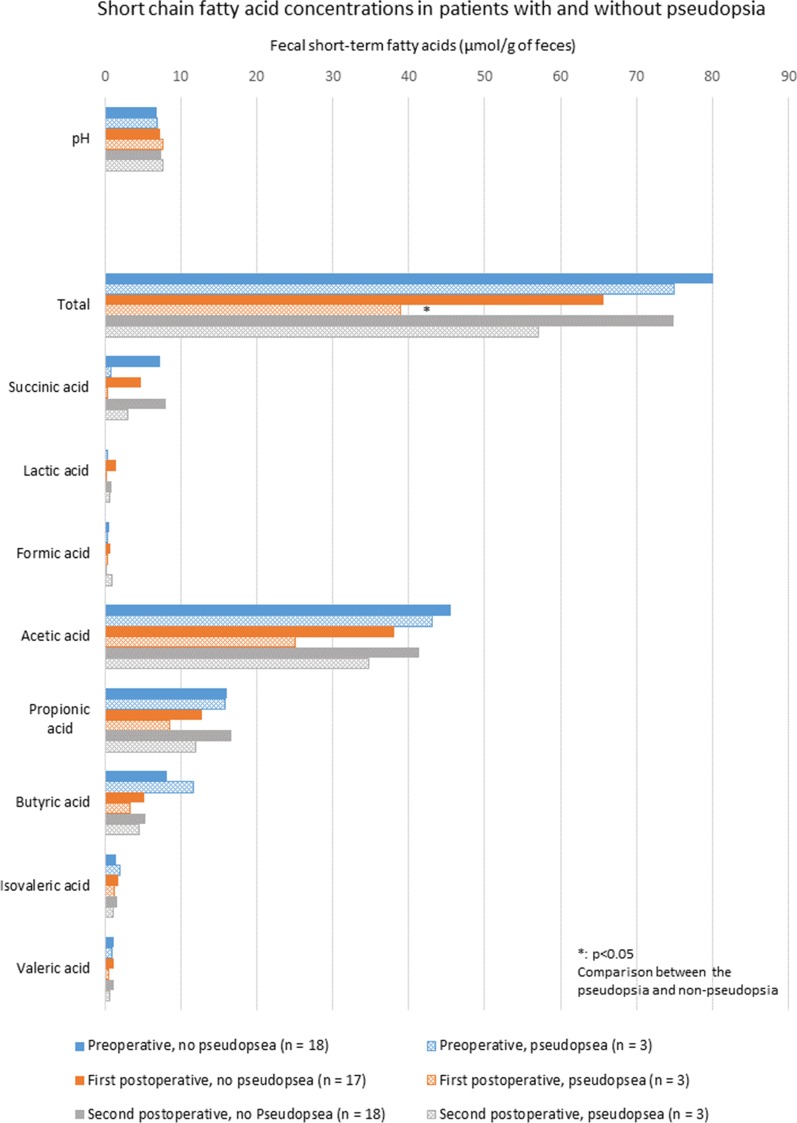
Perioperative changes in fecal pH and concentrations of short-chain fatty acids in feces between patients with and without pseudopsia. Blue bars represent preoperative values, orange bars represent first postoperative values, and gray bars represent second postoperative values (around POD 7). The mesh pattern depicts the pseudopsia group. For each measurement timepoint, differences between patients with and without pseudopsia were compared using the t test. Asterisk shows significant differences. *POD* postoperative day

Microbiota counts and fecal SFCAs concentrations of the pseudopsia group were compared with those of the non-pseudopsia group (Figs. 3, 4). Postoperative *Staphylococcus* counts in the pseudopsia group were significantly higher than in the non-pseudopsia group for first postoperative fecal samples (3.2 ± 1.3 vs. 5.4 ± 0.9; *p* = 0.012). Similarly second postoperative fecal samples had higher *Staphylococcus* counts than in the non-pseudopsia group (5.3 ± 0.4 vs. 3.8 ± 1.1; *p* = 0.034). Postoperative *Pseudomonas* counts were significantly higher in the pseudopsia group than in the non-pseudopsia group for first postoperative fecal samples (4.0 ± 2.3 vs. 1.7 ± 0.7; *p* = 0.001) and second postoperative fecal samples (4.6 ± 2.7 vs. 1.7 ± 0.8; *p* = 0.001). Postoperative *Enterobacteriaceae* counts in first postoperative fecal samples were higher in the pseudopsia group than in the non-pseudopsia group (6.3 ± 0.7 vs. 7.2 ± 0.6; *p* = 0.001). *Atopobium* cluster counts were only significantly different across groups for preoperative fecal samples. Preoperative *Atopobium* cluster counts of the pseudopsia group were significantly lower than those of the non-pseudopsia group (6.8 ± 3.8 vs. 9.0 ± 0.7; *p* = 0.001). First postoperative fecal sample concentrations of total fecal SFCAs in the pseudopsia group were significantly lower than in the non-pseudopsia group (38.9 ± 8.5 vs. 65.7 ± 17.1 μmol/g of feces; *p* = 0.018).

### Microbiota counts and concentrations of fecal SFCAs in patients with and without insomnia

In preoperative fecal samples, there were significant differences in counts of *Enterobacteriaceae* (insomnia group, 5.5 ± 1.9 log_10_ cells/g of feces; non-insomnia group, 6.9 ± 0.5 log_10_ cells/g of feces; *p* = 0.044) and *Enterococcus* (insomnia group: 5.5 ± 1.8; non-insomnia group, 6.9 ± 1.4; *p* = 0.049). However, there were no significant postoperative differences. There were also no significant differences in concentrations of fecal SFCAs in patients with versus without insomnia.

## Discussion

This study revealed changes in the gut microbiota after cardiac surgery that persisted for 1 week even when patients restarted oral intake just after surgery. Total bacterial counts were significantly lower in the first and second postoperative samples, as well as both obligate anaerobe and facultative anaerobe counts. Fecal pH was significantly higher in the first and second postoperative fecal samples. In patients with pseudopsia, the first and second postoperative fecal samples had significantly higher *Staphylococcus* and *Pseudomonas* counts. Total fecal SFCAs concentrations were significantly lower in the first postoperative fecal samples.

In a previous study, obligatory anaerobe counts in patients with systemic inflammatory response syndrome were significantly lower than in normal subjects. Fecal pH was higher and fecal organic acid concentrations were dramatically lower in such patients [10]. In this study, similar findings were observed postoperatively. Cardiac surgery with cardiopulmonary bypass causes systemic inflammation [3, 8]. In addition, patients in our study, generally fasted on the day of surgery and received antibiotics and anesthetic agents perioperatively. Antibiotics cause damage to the gut flora [10, 22]. In this study, microbiota counts after cardiac surgery were significantly lower and gut flora components changed.

Postoperative *Lactobacillus* counts were significantly lower. *Lactobacillus* are a major component of the gut flora. Many studies have investigated the relationship between *Lactobacillus* counts and disease [21, 22]. Lower *Lactobacillus* counts have been observed in irritable bowel syndrome, type 1 diabetes, and multiple sclerosis but higher counts have been observed in colon disease and rheumatoid arthritis. The clinical implications of a lower *Lactobacillus* count after cardiac surgery are not immediately apparent. Decreases in total microbiota counts may have important clinical implications. The vaginal microbiota is characterized by low diversity and *Lactobacillus* colonization, which may play an important role in microbiota hemostasis [23].

SCFAs including butyrate, propionate, and acetate play key roles in gut barrier function [24], gut motility, and immune response [25]. SCFAs, which have neuroactive properties, might be directly or indirectly involved in communication along the microbiota–gut–brain axis [26]. SCFAs have been implicated in a range of neuropsychiatric disorders, such as Parkinson disease, Alzheimer disease, and depression [27]. Fecal SCFA concentrations are lower in patients with depression than in controls [28]. Furthermore, butyrate administration was associated with recovery of memory function and increased expression of genes implicated in associative learning in a mouse model of Alzheimer disease [29]. SCFAs may be associated with postoperative mental disorder. A significant decrease in total fecal acid concentrations might be associated with postoperative delirium. Unfortunately, in this study, the number of patients with postoperative delirium may be too small to identify any associations between SCFA concentrations and postoperative delirium. However, postoperative fecal pH was significantly higher than preoperative pH, which might be associated with postoperative mental disorder. Bacteria which secrete SCFA, such as *Lactobacillus* decreased postoperatively, suggesting microbiome was damaged and led to leaky gut syndrome and bacterial translocation. Consequently, central nerve system may be influenced.

Antibiotics have a significant impact on the gut microbiota [30]. Oral or intravenous antibiotic use might reduce the gut microbiota. Furthermore, Kohler et al. reported that infections and antibiotics are associated with a risk of severe mental disorders [31]. Some reports have claimed that probiotic intake reduces scores on the Depression Anxiety Stress Scale [6]. Antibiotics could modulate components of the gut microbiota possibly, resulting in mental disorders.

Higher *Staphylococcus* and *Pseudomonas* counts were observed in patients with pseudopsia in this study. A previous study reported that the peak *Staphylococcus* and *Enterococcus* count were associated with the risk of enteritis in patients with systemic inflammatory response syndrome [32]. *Pseudomonas* is an important pathogen [33]. *Pseudomonas* in the gut flora increases very quickly in severely ill patients [34]; increased *Pseudomonas* count is associated with septic complications in patients with systemic inflammatory response syndrome [32]. Oral probiotic intake reduces the incidence of *Pseudomonas* infections [35]. Damaged gut flora could cause leaky gut syndrome, which is associated with bacterial translocation or bacterial toxins translocation [36]. Lipopolysaccharide (LPS) is a component of gram-negative bacteria such as *Pseudomonas*.[37] LPS translocation could be occurring in leaky gut syndrome. LPS could evoke strong systemic inflammation [38, 39]. Increased gram-negative bacteria levels in the gut flora could increase LPS translocation. Some past studies have reported that brain damage is related to gut dysbiosis or endotoxemia [38, 40]. Increased *Pseudomonas* counts might be related to more severe illness. However, it is not clear from this study whether increased *Pseudomonas* counts are directly related to postoperative pseudopsia. Further study is necessary to understand the relationship.

There were some limitations to this study. The most obvious limitation in this research was that of a small sample size. Also, before 21 study participants were recruited, 23 potential participants declined to participate because the study design imposes a burden on each participant just after cardiac surgery. Thus, selection bias was likely present. Although *Lactobacillus* preparations are very popular in Japan, habitual *Lactobacillus* preparation intake among 47.6% of patients seems high. Patients with interest in *Lactobacillus* might have been more likely to participate.

Moreover, there were no patients with delirium among 21 patients after on-pump cardiac surgery, which was not the case in previous studies [1, 41]. This might be due to severely ill patients not meeting the study eligibility criteria. The assessment of delirium was based on the CAM-ICU, which is a categorical, not quantitative, diagnosis. It was possible that some cases of hypoactive delirium were not detected. Pseudopsia is considered to be a symptom of delirium that may be associated with the pathophysiology of delirium [13, 42].

Even if some limitations existed, this study is valuable in that the gut flora after cardiac surgery was investigated with fecal samples instead of rectal swabs. This is the pivot study to avoid the risk of statistical analysis standard error for small population. This allowed for objective evaluation of microbiota counts and fecal organic acid concentrations. In the field of postoperative cardiac care, induction of symbiotic therapy is considered to be easier after cardiac surgery than after abdominal surgery because the gastrointestinal tract is not damaged directly. Further study is necessary to verify the usefulness of perioperative cardiac symbiotic therapy.

## Conclusion

The gut microbiota can be damaged by cardiac surgical procedures, antibiotics and fasting, and the damage can persist for more than 1 week. Furthermore, Higher *Staphylococcus* and *Pseudomonas* counts were observed in patients with pseudopsia.

## Abbreviations

LPS: Lipopolysaccharide
qRT-PCR: Quantitative reverse transcription polymerase chain reaction
POD: Postoperative days
ICU: Intensive care unit
SCFAs: Short-chain fatty acids concentrations
